# A bronchoprotective role for *Rgs2* in a murine model of lipopolysaccharide-induced airways inflammation

**DOI:** 10.1186/s13223-018-0266-5

**Published:** 2018-10-01

**Authors:** Tresa George, Mainak Chakraborty, Mark A. Giembycz, Robert Newton

**Affiliations:** 1 Airways Inflammation Research Group, Snyder Institute for Chronic Diseases, University of Calgary, Calgary, AB T2N 4Z6 Canada; 2 Immunology Research Group, Snyder Institute for Chronic Diseases, University of Calgary, Calgary, AB T2N 4Z6 Canada

**Keywords:** Inflammation, Regulator of G-protein signaling, RGS2, Bronchoconstriction, Lipopolysaccharide, Exacerbation, Murine

## Abstract

**Background:**

Asthma exacerbations are associated with the recruitment of neutrophils to the lungs. These cells release proteases and mediators, many of which act at G protein-coupled receptors (GPCRs) that couple via Gq to promote bronchoconstriction and inflammation. Common asthma therapeutics up-regulate expression of the regulator of G protein signalling (RGS), RGS2. As RGS2 reduces signaling from Gq-coupled GPCRs, we have defined role(s) for this GTPase-activating protein in an acute neutrophilic model of lung inflammation.

**Methods:**

Wild type and *Rgs2*^−*/*−^ C57Bl6 mice were exposed to nebulized lipopolysaccharide (LPS). Lung function (respiratory system resistance and compliance) was measured using a SCIREQ flexivent small animal ventilator. Lung inflammation was assessed by histochemistry, cell counting and by cytokine and chemokine expression in bronchoalveolar lavage (BAL) fluid.

**Results:**

Lipopolysaccharide inhalation induced transient airways hyperreactivity (AHR) and neutrophilic lung inflammation. While AHR and inflammation was greatest 3 h post-LPS exposure, BAL neutrophils persisted for 24 h. At 3 h post-LPS inhalation, multiple inflammatory cytokines (CSF2, CSF3, IL6, TNF) and chemokines (CCL3, CCL4, CXCL1, CXCL2) were highly expressed in the BAL fluid, prior to declining by 24 h. Compared to wild type counterparts, *Rgs2*^−*/*−^ mice developed significantly greater airflow resistance in response to inhaled methacholine (MCh) at 3 h post-LPS exposure. At 24 h post-LPS exposure, when lung function was recovering in the wild type animals, MCh-induced resistance was increased, and compliance decreased, in *Rgs2*^−*/*−^ mice. Thus, *Rgs2*^−*/*−^ mice show AHR and stiffer lungs 24 h post-LPS exposure. Histological markers of inflammation, total and differential cell counts, and major cytokine and chemokine expression in BAL fluid were similar between wild type and *Rgs2*^−*/*−^ mice. However, 3 and 24 h post-LPS exposure, IL12B expression was significantly elevated in BAL fluid from *Rgs2*^−*/*−^ mice compared to wild type animals.

**Conclusions:**

While *Rgs2* is bronchoprotective in acute neutrophilic inflammation, no clear anti-inflammatory effect was apparent. Nevertheless, elevated IL12B expression in *Rgs2*^−*/*−^ animals raises the possibility that RGS2 could dampen Th1 responses. These findings indicate that up-regulation of RGS2, as occurs in response to inhaled corticosteroids and long-acting β_2_-adrenoceptor agonists, may be beneficial in acute neutrophilic exacerbations of airway disease, including asthma.

**Electronic supplementary material:**

The online version of this article (10.1186/s13223-018-0266-5) contains supplementary material, which is available to authorized users.

## Background

Stable allergic asthma is characterized by airways inflammation, pulmonary eosinophilia, airways hyperreactivity (AHR) and mucus hypersecretion. In contrast, exacerbations of asthma involve worsening AHR and are associated with pulmonary neutrophilia [1]. In inflammation, mediators including histamine, leukotrienes, certain prostaglandins and possibly acetylcholine, are produced by multiple cell types, including neutrophils, epithelial cells, airways smooth muscle (ASM) and nerves. By acting on G protein-coupled receptors (GPCRs) that signal through the heterotrimeric G protein, Gq, and which are present on ASM, these mediators may promote bronchoconstriction [2]. However, agonists at Gq-coupled GPCRs can also enhance the expression of inflammatory cytokines and could therefore contribute towards increased inflammation [3, 4]. Similarly, many allergens, for example house dust mite (HDM) or cockroach allergen, are major triggers of asthma and contain proteolytic activities that activate a family of GPCRs known as protease-activated receptors (PARs) [5, 6]. PARs may signal via multiple transducers, which include Gq, as well as Gi, G12/13, and likely β-arrestin, and may contribute to various inflammatory responses [5–7] Thus, HDM extracts produce profound airway inflammation and AHR in mouse models of asthma [8–10]. Indeed, many proteases, including those in HDM or cockroach allergen, elicit inflammatory responses and, in vivo, may act via PAR2, which can couple via Gq and other transducers [7, 11], to induce inflammation and reduce lung function [12–15]. Similarly, activation of coagulation cascades during inflammation can activate PAR1 to up-regulate inflammatory cytokine expression and PAR2 deficiency produced increased expression of the C-X-C motif chemokine ligand (CXCL), CXCL1, following intratracheal instillation of the Gram-negative bacterial well-wall component, lipopolysaccharide (LPS) [16–18]. Likewise, neutrophil recruitment and activation leads to release of elastase, and other proteases, that can activate both PAR1 and PAR2 to promote cytokine expression and inflammation [19–21]. Indeed, inhibition of neutrophil elastase improved AHR and inflammation in a mouse model of airways inflammation, suggesting that neutrophil-derived proteolytic activity can be a major driver of inflammation [22].

The regulator of G protein signaling (RGS) family of proteins interact with active GTP-bound, Gα, to promote intrinsic GTPase activity and GTP hydrolysis [23]. This returns the heterotrimeric G protein to an inactive heterotrimeric (αβγ) GDP-bound state and switches off GPCR signaling. Pertinent to the current study are the R4 sub-family of RGS proteins as these have selectivity for Gαq (and Gαi) [23, 24]. While many R4 family members, including RGS2-5 are expressed at the mRNA level in human ASM [25], loss of *Rgs2*, *Rg*s4 and *Rgs5* in the mouse produce AHR and/or enhanced ASM contractility [25–30]. Of relevance to therapeutics used in asthma is the finding that RGS2 mRNA is induced in vivo in human airways following budesonide inhalation [31]. Furthermore, RGS2 mRNA and protein are increased by β_2_-adrenoceptor agonists in a manner that is synergistically enhanced by glucocorticoids in human ASM [26]. Similar effects also occur in primary bronchial epithelial cells, where RGS2 is more glucocorticoid-inducible [32].

While RGS2 is bronchoprotective and its expression may be enhanced by commonly used asthma therapeutics, expression of RGS2 in non-contractile cells suggests additional roles in the airways. Indeed, in airway epithelial cells, signaling and cytokine expression induced by agonists of Gq-coupled GPCRs, including the histamine H_1_, muscarinic M_3_ and thromboxane receptors was reduced by RGS2 [32]. As PARs are Gq-coupled GPCRs present on the airway epithelium and promote inflammatory cytokine expression [33, 34], their targeting by RGS2 could be therapeutically beneficial. Indeed, a PAR1 antagonist markedly reduced neutrophil influx into the mouse lung 4 and 24 h post infection with *S. pneumonia* [35]. Given that PARs mediate inflammatory responses following their cleavage by proteases released by neutrophils and other inflammation-activated processes [7, 36], we used a mouse, LPS-driven, model of airway inflammation and AHR to mimic the effects of acute bacterial infection. This allows effects of *Rgs2* deficiency to be explored in an acute neutrophilic setting [37], and which may be relevant to exacerbations of airway diseases, including asthma [1].

## Methods

### Nomenclature

Unless indicated, genes, mRNA or protein are referred to using official gene symbols as provided by The National Center for Biotechnology Information (NCBI; https://www.ncbi.nlm.nih.gov/). Mouse genes are shown in lower case, but italicised with an upper case first letter, whereas the expressed products (protein and, for consistency, mRNA) are written as uppercase non-italicised letters, as per the Mouse Genome Informatics website (http://www.informatics.jax.org/).

### Animals and treatment protocols

Wild type female C57BL/6 mice were purchased from Charles River Laboratories (Wilmington, MA, USA) and kept in the Clara Christie Centre for Mouse Genomics at the University of Calgary prior to use at ages 10–12 weeks. Alternatively, *Rgs2* wild-type, and knockout (*Rgs2*^−/−^) mice, on a C57BL/6 background, were bred in-house through *Rgs2*^+/−^ × *Rgs2*^+/−^ crosses for use at ages 10–12 weeks. These animals were previously re-derived from mice donated by Dr. Scott Heximer (University of Toronto) [38, 39]. Ear punches were used for genotyping following DNA isolation by the MicroLYSIS protocol (Gel Company, Inc.; San Francisco, CA). TaqMan PCR (Applied BioSystems; Foster City, CA) was performed using the allele-specific primers and probes (Additional file 1: Table S1). For the LPS exposures, mice were placed in a closed plexiglass chamber connected to a nebulizer and 20 ml LPS (*E. coli* 0127:B8) at 150 μg/ml in phosphate buffered saline (PBS) was aerosolized over a period of 30 min. Control mice were exposed to 20 ml aerosolized PBS over the same timeframe. LPS-exposed mice were collected for assessment of lung function and lung inflammation at 3, 6 and 24 h following inhalation. PBS-exposed mice were collected 3 h after exposure for all measurements. Protocols were approved by University of Calgary Animal Care Committee according to the Canadian Council for Animal Care guidelines. Mice were age and sex matched between the different genotype groups and treatments.

### Lung function analysis

Mice were weighed and anesthetized by intraperitoneal injection of sodium pentobarbital (50 mg/kg) (CEVA Santé Animale, Libourne, France) and intramuscular injection of ketamine hydrochloride (90 mg/kg) (Wyeth, St Laurin, QC, Canada). Anesthetized animals were tracheostomized and connected to a SCIREQ flexivent small animal ventilator (SciReq, Montreal, Ontario). Animals were challenged with aerosolized PBS followed by increasing half-log concentrations (3–300 mg/ml) of methacholine (MCh) made up in PBS administered via an in-line nebulizer. Measurement of respiratory system resistance (resistance) and compliance was performed using a snapshot 150 perturbation, as described [40].

### Immunohistochemistry

The left lung was inflated and fixed with buffered 10% formalin, and then embedded in wax. Serial, 4 μm, sections were adhered to positively charged slides (Fisher Scientific, Nepean, Ontario, Canada) prior to staining with either hematoxylin and eosin (H&E) or periodic acid-schiff (PAS). Slides were visualised by light microscopy using an Olympus IX51 microscope (Olympus Canada Inc, Richmond Hill, Ontario, Canada). Images were captured with an Olympus Q color 5 charge-coupled device camera (model RTV-R-CLR-10) with Q-Capture Pro acquisition imaging software.

Hematoxylin and eosin stained sections were analyzed using a semi-quantitative scoring system to evaluate the fraction of the airway that was occupied by inflammatory cell infiltrates: 4 = robust inflammation (more than 50% of airway circumference surrounded by inflammatory cell infiltrates); 3 = moderate inflammation (25–50% of airway circumference surrounded by inflammatory cell infiltrates); 2 = mild inflammation (10–25% of airway circumference surrounded by inflammatory cell infiltrates); 1 = minimal inflammation (< 10% of airway circumference surrounded by inflammatory cell infiltrates) and a score of 0 = no inflammatory cell infiltrates. Numerical data were averaged from 3 sections for each animal. ASM thickness was measured on H&E stained sections at 4 points around the circumference of a bronchiole by constructing a line perpendicular to the airway circumference and using Image J software to measure the distance from the outer edge to the inner edge of the ASM layer. An average of the 4 measurements was recorded for one bronchiole/section. All measurements were made by an investigator blinded to the study treatments.

PAS-stained sections were scored using a semi-quantitative system where a value of 4 indicated strong staining (more than 75% of the airway epithelium PAS positive), 3 = moderate staining (50–75% of the airway epithelium PAS positive), 2 = mild staining (25–50% of the airway epithelium PAS positive), and a score of 1 = minimal staining (less than 25% of airway epithelium PAS positive). Numerical data were averaged from three sections from each animal. All measurements were analysed by an investigator blinded to the tissue codes.

### BAL fluid cell counts and cytokine release

Lungs were lavaged with 3 × 0.5 ml of ice cold PBS. Following centrifugation, BAL fluid was frozen at − 80 °C for later analysis of cytokines. Pelleted cells were resuspended in 1 ml PBS for total cell counting. Cells were also spun onto glass slides prior to staining with Diff-Quik (Canadawide, Ottawa, ON). Differential cell counts were performed on at least 400 cells/slide and macrophage, eosinophil, neutrophil and lymphocyte percentages converted to absolute numbers using the respective total cell counts. Cytokines/chemokines in the BAL fluid were quantified with a mouse cytokine/chemokine multiplex Luminex Array (Eve Technologies, Calgary, AB, Canada). The limit of detection for most analytes was 0.64–3.2 pg/ml. When an analyte was not detected, the lower detection limit was reported.

### RNA extraction and real-time PCR

Frozen right lung lobe tissue was disrupted in a TissueLyser LT (Qiagen; Valencia, CA) and RNA prepared using RNeasy Plus kits (Qiagen). Reverse-transcription was performed on 0.5 µg total RNA with qScript kits (Quanta: Gaithersburg, MD). Real-time PCR was performed using SYBR GreenER (Life Technologies; Burlington, OH) chemistry and the primers listed in Additional file 1: Table S2 with either a StepOne Plus or a 7900HT PCR instrument (Applied BioSystems). Samples were analyzed in duplicate. Dilution of cDNA was used to generate standard curves for target genes. Relative expression data were normalized to the housekeeping gene GAPDH.

### Data presentation and statistical analysis

Data from *N* animals are expressed as mean ± SE. Graphing and statistics were performed using GraphPad Prism v6 (La Jolla, CA). Significance was tested as described in figure legends. Where possible, and as appropriate, parametric analyses were used. The null hypothesis was rejected when: *P < 0.05, **P < 0.01, ***P < 0.001. Normality testing was performed using the D’Agostino & Pearson omnibus normality test in GraphPad Prizm v6.

## Results

### Effect of LPS on lung function in wild type and *Rgs2*^−/−^ mice

**Fig. 1:**
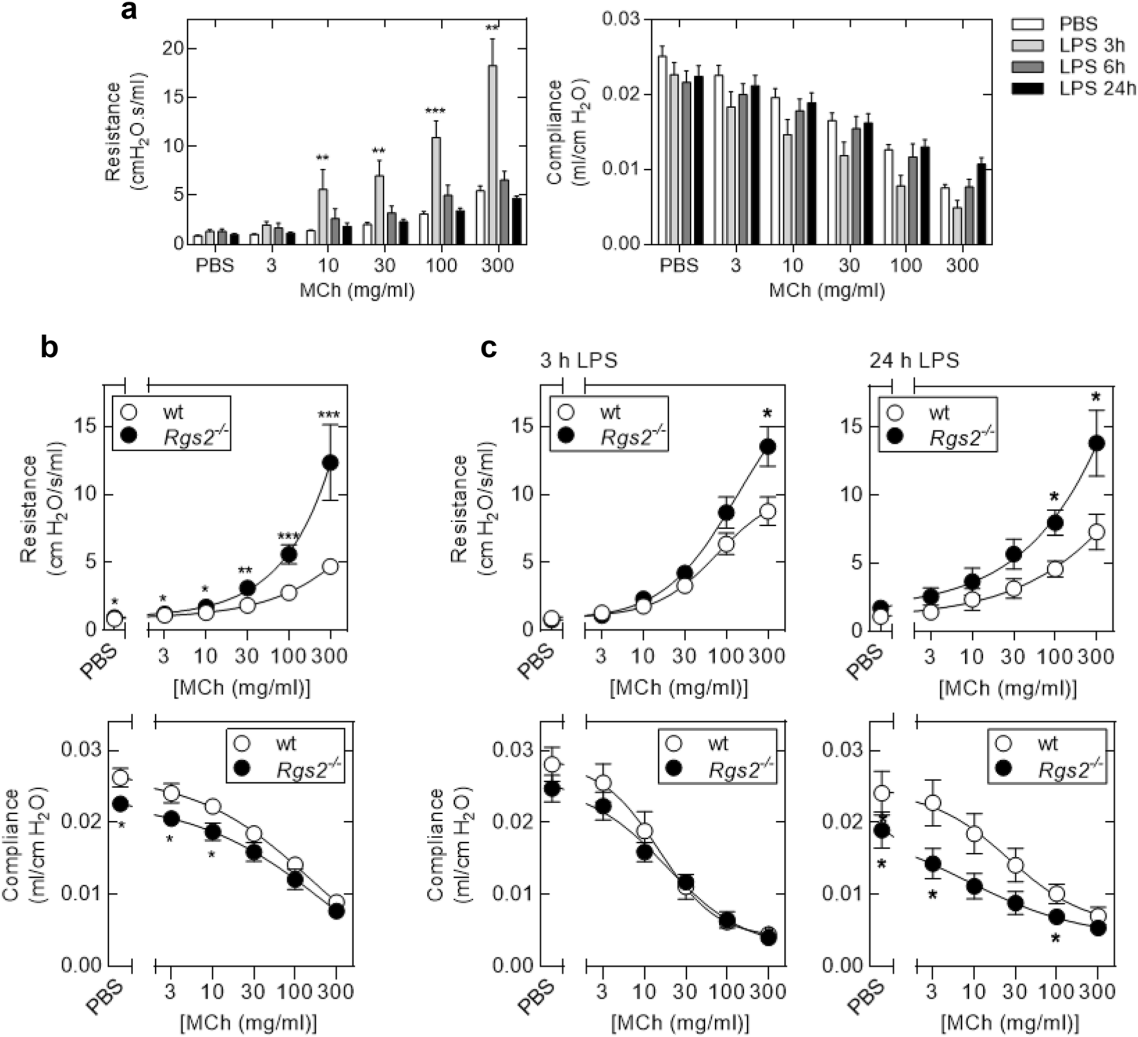
Effect of LPS exposure on lung function in wild type and *Rgs2* deficient animals. **a** Wild type mice were exposed to aerosolized PBS (20 ml) or LPS from *E. coli* 0127:B8 (20 ml of 150 μg/ml) for 30 min. The mice were attached to a flexivent apparatus and lung function was analysed, at 3, 6 or 24 h following LPS exposure. Respiratory system resistance (Resistance) and compliance were determined using a snapshot 150 perturbation following inhaled challenge with nebulized PBS (10 µl) and methacholine (MCh) (10 µl of 3, 10, 30, 100, 300 mg/ml). Data (*N* = 11–13) are plotted as cm H_2_O/s/ml (resistance) or ml/cm H_2_O (compliance) as mean ± SE. Significance was tested against PBS control using ANOVA with a Dunn’s post test. **P < 0.01, ***P < 0.001. **b** Wild type (wt) (*N* = 6) and *Rgs2*^−/−^ (*N* = 3) mice were exposed to aerosolized PBS (20 ml) for 30 min prior to lung function analysis at 3 h. Resistance and compliance were measured following inhaled challenge with nebulized PBS and PBS containing increasing concentrations of MCh. To enable definitive analysis of *Rgs2* loss in naïve (non-inflamed) animals, the current data were combined with that for previously analysed PBS exposed/naïve mice [26, 28]. Data (wild type, *N* = 26; *Rgs2*^−*/*−^, *N* = 23) are plotted as cm H_2_O/s/ml (resistance) or ml/cm H_2_O (compliance) as mean ± SE. Significance between wild type and *Rgs2*^−*/*−^ animals was tested by Mann–Whitney U test. *P < 0.05, **P < 0.01, ***P < 0.001. **c** Wild type (wt) and *Rgs2*^−/−^ mice were exposed to aerosolized PBS or LPS from *E. coli* 0127:B8 (20 ml at a concentration of 150 μg/ml) for 30 min and subjected to lung function analysis 3 or 24 h post-LPS exposure. Resistance and compliance was measured following inhalation of nebulized PBS and PBS containing increasing concentrations of MCh. Data (*N* = 6–7) are plotted as cm H_2_O/s/ml (resistance) or ml/cm H_2_O (compliance) as mean ± SE. Significance between wild type and *Rgs2*^−*/*−^ animals was tested by Mann–Whitney U test. *P < 0.05

In initial experiments, wild type C57Bl/6 mice were exposed to aerosolized PBS (20 ml) or LPS (20 ml at 150 μg/ml in PBS) administered over a 30 min period. Lung function was measured 3, 6 and 24 h following LPS exposure. Baseline resistance and compliance i.e. following PBS challenge, was not different between the LPS- and PBS-exposed animals (Fig. 1a). However, 3 h after LPS exposure, MCh challenge induced significantly greater increases in lung resistance when compared to the PBS-exposed animals (Fig. 1a). While corresponding losses in compliance were also produced, this did not reach statistical significance. By 6 and 24 h post-LPS exposure, these effects on lung function had returned to near baseline and no significant differences relative to PBS controls were apparent (Fig. 1a).

Breeding from heterozygous *Rgs2*^−*/*+^ pairs to produce wild type and *Rgs2*^−*/*−^ knockout littermates allowed lung function to be compared between wild type and *Rgs2*^−*/*−^ animals. In prior studies [26, 28], we found that control animals, without any pro-inflammatory insult, showed AHR due to *Rgs2* loss. In the current study, *N* = 6 wild type and *N* = 3 *Rgs2*^−*/*−^ animals were subjected to PBS exposure and lung function was examined. These data were consistent with prior findings, which when taken together provide a definitive statement as to the effect of *Rgs2* gene loss on lung function in control, non-inflamed, animals (Fig. 1b). Compared to wild type animals, this analysis shows a markedly enhanced increase in the resistance produced by MCh in *Rgs2*^−*/*−^ mice (Fig. 1b, upper panel). Similarly, baseline compliance is significantly reduced in the *Rgs2*^−*/*−^ animals relative to wild type and following MCh challenge, this compliance decreases further (Fig. 1b, lower panel). These data unequivocally confirm a bronchoprotective effect for the wild type *Rgs2* gene in the absence of airway inflammation. Three hours following LPS exposure, basal PBS challenged airway resistance was similar for wild type and *Rgs2*^−*/*−^ animals (Fig. 1c). Following increasing doses of aerosolized MCh, the LPS-exposed *Rgs2*^−*/*−^ animals displayed a significantly enhanced increase in resistance compared to LPS-exposed wild type animals (Fig. 1c). A similar effect was apparent 24 h post-LPS exposure where the wild type animals showed signs that lung resistance was improving, while resistance in the *Rgs2*^−*/*−^ animals was significantly higher following MCh challenge (Fig. 1c). Indeed, in the *Rgs2*^−*/*−^ animals the MCh-induced resistance was essentially unchanged compared to that observed 3 h post-LPS exposure. Three hours post-LPS exposure, baseline compliance, i.e. following PBS challenge, was modestly lower in the *Rgs2*^−*/*−^ animals. This did not reach significance and was similar between wild type and *Rgs2*^−*/*−^ animals for all doses of inhaled MCh. However, 24 h following LPS exposure, the PBS challenged, baseline compliance, and that following MCh challenge was significantly reduced in the *Rgs2*^−*/*−^ animals compared to wild type. Thus, while LPS produced a transient loss of lung function in wild type mice (Fig. 1a), the presence of wild type *Rgs2* was nevertheless protective (Fig. 1c). However, 24 h post-LPS exposure, while lung function in wild type animals was recovering, MCh-induced airway resistance remained high in the *Rsg2* deficient mice (Fig. 1c). Furthermore, 24 h post-LPS exposure, the lungs of the *Rgs2*^−*/*−^ animals were significantly stiffer (reduced compliance) than in the wild type counterparts (Fig. 1c).

### Inflammatory cell recruitment in wild type and *Rgs2*^−/−^ animals

**Fig. 2:**
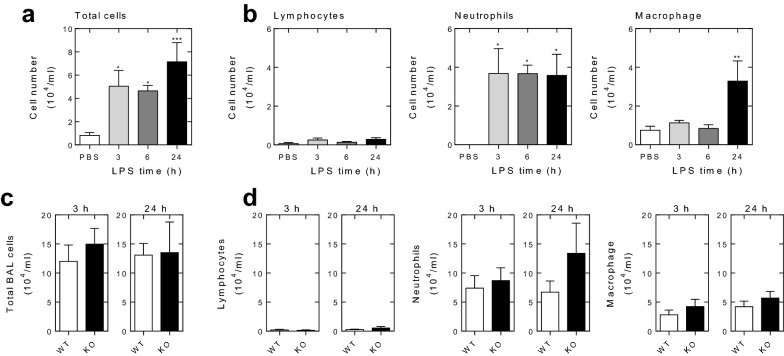
Effect of *Rgs2* deficiency on LPS-induced cell influx into bronchoalveolar lavage. Wild type mice were exposed to aerosolized PBS (20 ml) or LPS from *E. coli* 0127:B8 (20 ml of 150 μg/ml) for 30 min and bronchoalveolar lavage (BAL) was performed after 3, 6 or 24 h of LPS exposure. **a** Total cell counting in BAL fluid was performed. Data (*N* = 4–6) are plotted as mean ± SE. Significance compared against PBS control was assessed by ANOVA with a Dunnett’s post test. *P < 0.05, ***P < 0.001. **b** Following DiffQuick staining of cytospin slides, differential cell counting was performed. Data (*N* = 4–6) are plotted as mean ± SE. Significance compared with PBS control was assessed by ANOVA with a Dunn’s post test. *P < 0.05, **P < 0.01. **c** Wild type (WT) and *Rgs2*^−/−^ (KO) mice were exposed to aerosolized LPS, as above. The BAL fluid was collected after 3 and 24 of LPS treatment for counting of total cell numbers and the data (*N* = 6–7) plotted as mean ± SE. **d** After Diff-Quick staining of cytospin slides, differential cell counting was performed and the data (*N* = 4–7) plotted as mean ± SE. In C and D, significance was tested between WT and KO using Mann–Whitney U test. No significant differences were found

Analysis of total inflammatory cells present in the BAL fluid obtained from PBS exposed mice revealed only low levels of inflammatory cells that were predominantly made up of macrophage (Fig. 2a, b). In contrast, at each time, 3, 6 and 24 h, following LPS exposure, there was a dramatic and significant increase in total inflammatory cells recruited into the lungs (Fig. 2a). While differential cell counting showed very modest and non-significant increases in lymphocytes, the number of neutrophils, which were essentially absent in PBS exposed animals, was dramatically increased at all times following LPS exposure (Fig. 2b). Such data are as previously described [37]. Compared to PBS exposure, the number of macrophage remained essentially unaltered at 3 and 6 h post-LPS, but were significantly elevated at 24 h (Fig. 2b). Thus, at baseline, following PBS exposure, macrophage accounted for essentially all the inflammatory cells present in the BAL fluid, but following LPS inhalation, there was a rapid influx of neutrophils. These remained elevated at 3, 6 and 24 h and accounted for the majority of the inflammatory cells present in the airways. At 24 h, there was a significant increase, compared to PBS control, in macrophage and this explains the slightly elevated total cells counts observed at this time (Fig. 2a, b).

In prior studies, we found no differences in the total or differential BAL fluid cell counts between naïve, non-inflamed, wild type and *Rgs2*^−*/*−^ animals [26, 28]. In each case, wild type or *Rgs2*^−*/*−^ animals, the total cell counts were essentially identical and consisted of macrophage. In the current study, total and differential cell counting was therefore not repeated on the naïve/control animals. However, comparison between wild type and *Rgs2*^−*/*−^ animals exposed to LPS for either 3 or 24 h also revealed no differences in the total cells counts (Fig. 2c). Similarly, differential cell counting revealed no differences between the lymphocyte, neutrophil or macrophage numbers recruited to the airways of wild type and *Rgs2*^−*/*−^ animals at either time (Fig. 2d).

### Effect of *Rgs2* deficiency on LPS-induced lung inflammation

**Fig. 3:**
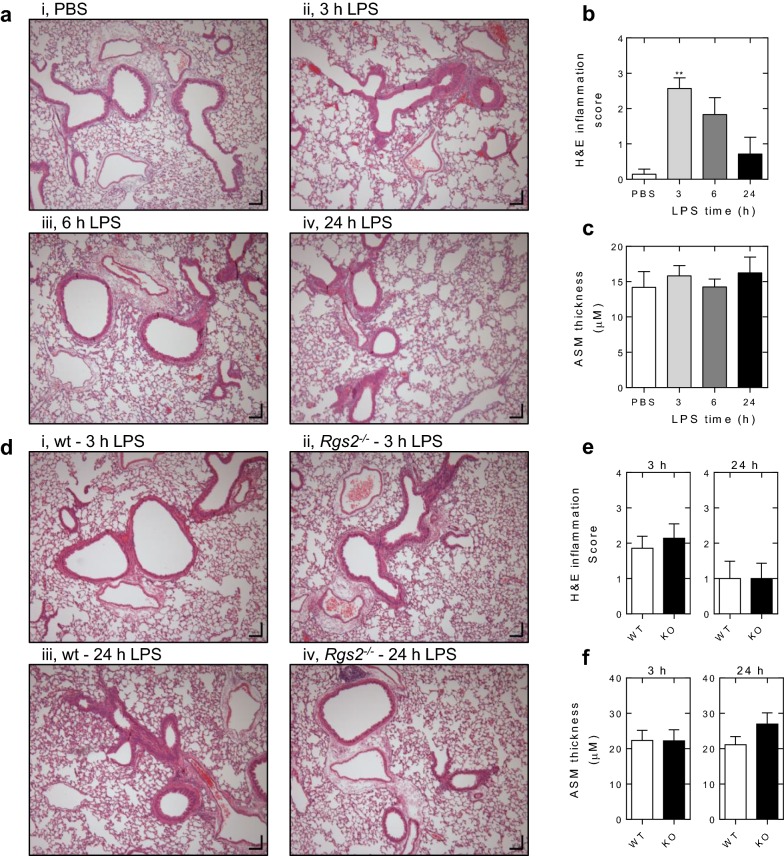
Effect of *Rgs2* deficiency on LPS-induced lung inflammation. Wild type mice were exposed to aerosolized PBS (20 ml) or LPS from *E. coli* 0127:B8 (20 ml at 150 μg/ml) for 30 min. The lungs were harvested 3, 6 or 24 h after LPS challenge and fixed for later sectioning. Lung sections were stained with H&E. **a** Representative H&E stained sections of left lung are shown at ×10 magnification. **b** The H&E stained sections were manually scored where: 0 = no inflammation around airways, 1 = (minimal) < 10% inflammation around airways, 2 = (mild) 10–25, 3: (moderate) 25–50%, 4: (high) > 50%. Data (*N* = 6–7) are plotted as mean ± SE. Significance relative to PBS control was assessed by ANOVA with a Dunn’s post test. *P* ≤ 0.01 (**). **c** Morphometric analysis of ASM thickness. Data (*N* = 8) are plotted in µm as mean ± S.E. **d** As in A, wild type (WT) and *Rgs2*^−/−^ (KO) mice were exposed to aerosolized LPS for 3 and 24 h prior to fixing, sectioning and H&E staining. Representative images are shown. **e** Scoring of H&E staining images was performed as in A. Data (*N* = 7) are plotted as mean ± SE. **f** ASM thickness was measured and data (*N* = 11–14) are plotted as mean ± SE. In **e** and **f**, significance was tested between WT and KO using Mann–Whitney U test. No significant differences were found

In wild type animals, LPS produced a rapid and profound increase in inflammatory cell infiltration around many of the airways and, often, the vessels (Fig. 3a). Scoring of H&E sections, revealed maximal cell influx to have occurred at 3 h, followed by a decline towards baseline levels at 24 h. Morphometric analysis of the H&E lung sections showed no effect of the LPS exposure on ASM thickness at any of these times (Fig. 3c). Prior studies [26, 28], showed there to be no differences between the lung inflammation scores of control/naïve wild type or *Rgs2*^−*/*−^ animals. In each case there was no evidence of inflammation and, therefore, this analysis was not repeated in the current study. Comparisons between the wild type and *Rgs2*^−*/*−^ animals showed no differences in lung inflammation, inflammation score, or on ASM thickness at either 3 or 24 h post LPS exposure (Fig. 3d–f).

### Effect of *Rgs2* deficiency on mucus production following LPS exposure

**Fig. 4:**
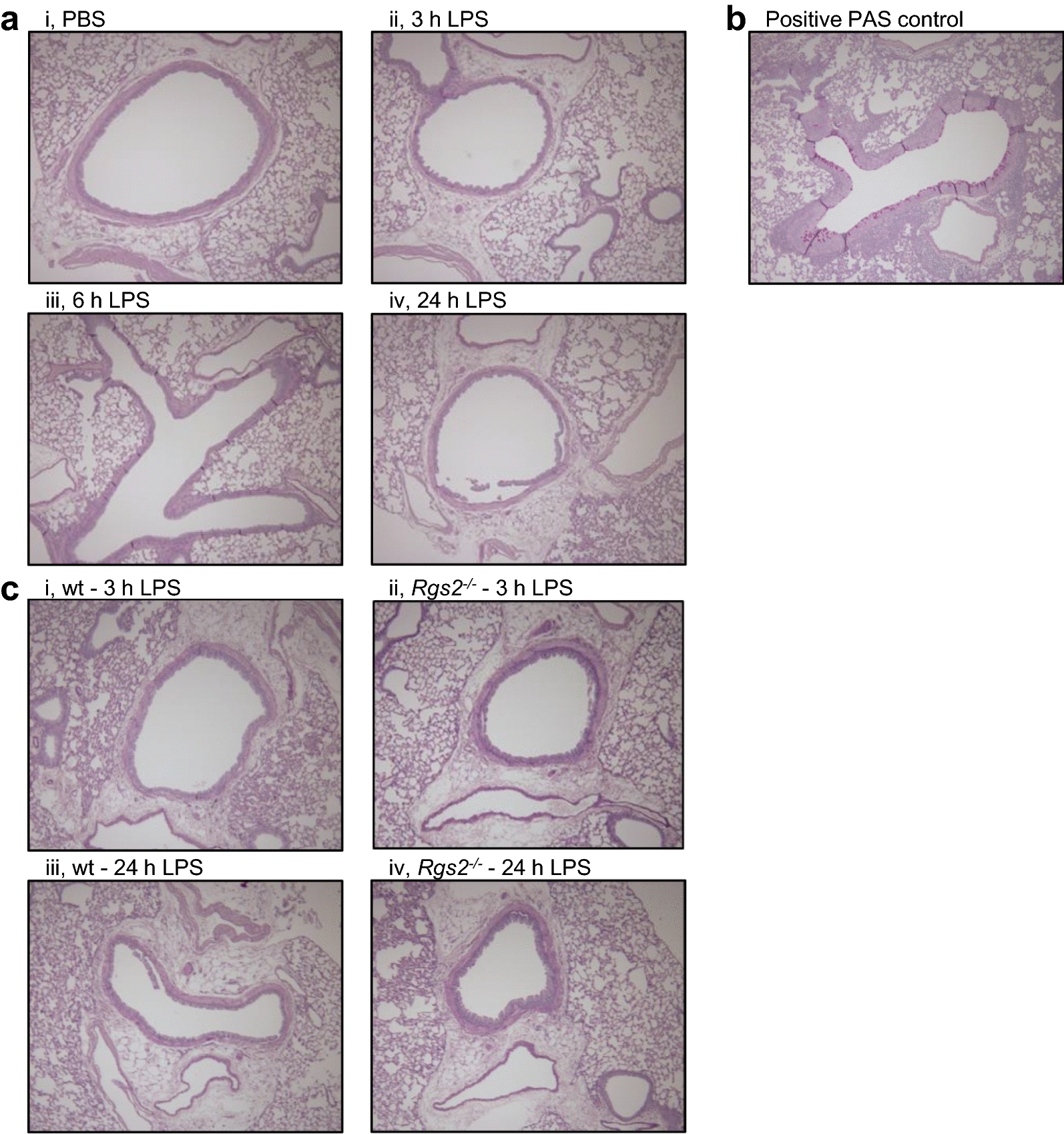
Effect of LPS and *Rgs2* deficiency on mucin production. Wild type mice were exposed to aerosolized PBS (20 ml) or LPS from *E. coli* 0127:B8 (20 ml at 150 μg/ml) for 30 min. The lungs were harvested 3, 6 or 24 h after LPS challenge and fixed for later sectioning. Lung sections were stained with H&E. **a** PAS stained sections of left lung are shown at ×10 magnification. Images are representative of at least six animals, all of which were negative for PAS. **b** Positive control for PAS staining. Wild type animals were exposed to *intra nasal* house dust mite exposure at 25 µg, 3× per week for 3 weeks prior to fixing and PAS staining. **c** Wild type (WT) or *Rgs2*^−/−^ (KO) mice were exposed to aerosolized LPS for 3 and 24 h prior to fixing, sectioning and PAS staining. Images representative of at least six animals are shown. While no PAS staining was apparent, positive control sections, as in **b**, produced marked PAS staining

Periodic acid-schiff staining was used to determine whether LPS promoted mucus secretion in murine lungs and the effect of *Rgs2* deficiency. As shown in Fig. 4a, there was no evidence of PAS staining 3, 6 or 24 h following LPS exposure. Positive control animals (intra-nasal HDM exposure at 25 µg, 3× per week for 3 weeks) [28], for which the sections were processed in parallel with the LPS-exposed sections, revealed robust PAS staining (Fig. 4b). These data, therefore, confirm that LPS did not induce mucus secretion over the 24 h following exposure. Similarly, prior analyses of control/naïve, non-inflamed, lungs from *Rgs2*^−*/*−^ animals also showed no evidence of PAS staining [26, 28]. Likewise, the analysis of PAS staining in wild type and *Rgs2*^−*/*−^ animals at 3 and 24 h following LPS exposure revealed no evidence of mucus secretion in either the wild type or *Rgs2*^−*/*−^ animals (Fig. 4c).

### Gene expression in the BAL fluid of LPS exposed wild type and *Rgs2*^−/−^ mice

BAL fluid from the experiments depicted in Fig. 2 was subjected to Luminex assay to determine the protein concentrations of cytokines, chemokines and growth factors. Many factors were at or below the limit of detection (typically ~ 0.5–3.2 pg/ml) for the Luminex assay. All analytes, including the classical Th1 cytokine, interferon γ (IFNG) and interleukins (IL) of the Th2 (IL4, IL5, IL13) and Th17 (IL17) cytokine families that fell below 5 pg/ml were deemed not to be present (Table 1A). For the majority of these factors there was no apparent modulation by LPS-exposure at either 3 or 24 h, or between wild type and *Rgs2*^−*/*−^ animals. However, within this lowly expressed group was CXCL9 (assay sensitivity = 0.49 pg/ml). This revealed an LPS-exposure induced expression of 2.1 ± 0.2 pg/ml at 3 h. This was significantly (*P* ≤ 0.01, Mann–Whitney U test) greater than for PBS treated of 0.7 ± 0.1 pg/ml, but was unaltered between wild type and *Rgs2*^−*/*−^ animals. More highly expressed factors, i.e. those showing peak expression of < 100 pg/ml are listed in Table 1B. A number of these, including the C-C motif ligands (CCL), CCL5 and CCL11, as well as CXCL10 and leukemia inhibitory factor (LIF), were significantly induced by LPS at 3 h post-exposure, but were again not modulated by *Rgs2* deficiency. The expression of IL12B showed a modest, but not significant, increase from 4.0 ± 1.0 to 8.6 ± 1.5 pg/ml 3 h following LPS exposure (Table 1B). This was significantly enhanced to 13.5 ± 1.6 pg/ml in the 3 h post-LPS *Rgs2*^−*/*−^ animals and this enhancement also persisted (*P* ≤ 0.001) 24 h post-LPS (Table 1B).

**Table 1:** Cytokines, chemokines and growth factors not detected or lowly expressed in the BAL fluid of LPS-exposed mice

A. Gene products not detected, or below 5 pg/ml, in BAL fluid	
Gene products	IFNG, IL2, IL3, IL4, IL5, IL10, IL12 (p70), IL13, IL17, CXCL9

Gene product	PBS	3 h post-LPS		24 h post-LPS	
	wt	wt	*Rgs2* ^−*/*−^	wt	*Rgs2* ^−*/*−^
B. Gene products showing low expression (< 100 pg/ml) in the BAL fluid following LPS exposure					
CCL2 (MCP-1)	7.9 ± 1.4	10.9 ± 1.7	11.3 ± 4.1	6.6 ± 1.1	5.3 ± 0.8
CCL5 (RANTES)	4.6 ± 0.6	10.1*** ± 0.9	8.8 ± 0.7	5.7 ± 0.3	5.1 ± 0.5
CCL11 (Eotaxin)	2.5 ± 0.3	10.2*** ± 1.6	10.7 ± 1.2	4.3 ± 1.1	4.8 ± 0.98
CSF1 (MCSF)	13.4 ± 9.5	5.9 ± 4.4	4.9 ± 3.1	1.4 ± 0.1	1.5 ± 0.2
CXCL5 (LIX)	38.6 ± 21.7	32.5 ± 17.9	79.2 ± 45.2	10.5 ± 7.4	10.7 ± 7.4
CXCL10 (IP10)	1.9 ± 0.3	59.9*** ± 12.9	45.5 ± 5.5	6.6 ± 1.3	5.0 ± 1.2
IL1A	20.4 ± 5.1	30.4 ± 4.4	30.1 ± 2.5	17.9 ± 2.8	24.4 ± 4.2
IL1B	2.0 ± 0.4	6.0 ± 1.5	8.3 ± 1.3	2.6 ± 0.6	2.6 ± 0.5
IL7	4.9 ± 1.2	5.4 ± 0.4	6.8 ± 0.7	3.9 ± 0.4	4.8 ± 0.2
IL9	14.9 ± 5.8	12.1 ± 1.4	13.4 ± 1.5	18.2 ± 3.8	22.5 ± 4.1
IL12B (IL12 p40)	4.0 ± 1.0	8.6 ± 1.5	13.47^# ^± 1.6	5.4 ± 0.7	12.79^### ^± 2.5
IL15	3.5 ± 0.6	6.4 ± 1.2	5.2 ± 1.1	2.7 ± 0.5	2.5 ± 0.5
LIF	0.7 ± 0.1	15.4** ± 1.7	19.0 ± 1.1	0.9 ± 0.1	0.9 ± 0.1
VEGF	8.0 ± 1.8	8.0 ± 2.3	8.3 ± 0.7	11.0 ± 3.1	11.0 ± 1.6

**Fig. 5:**
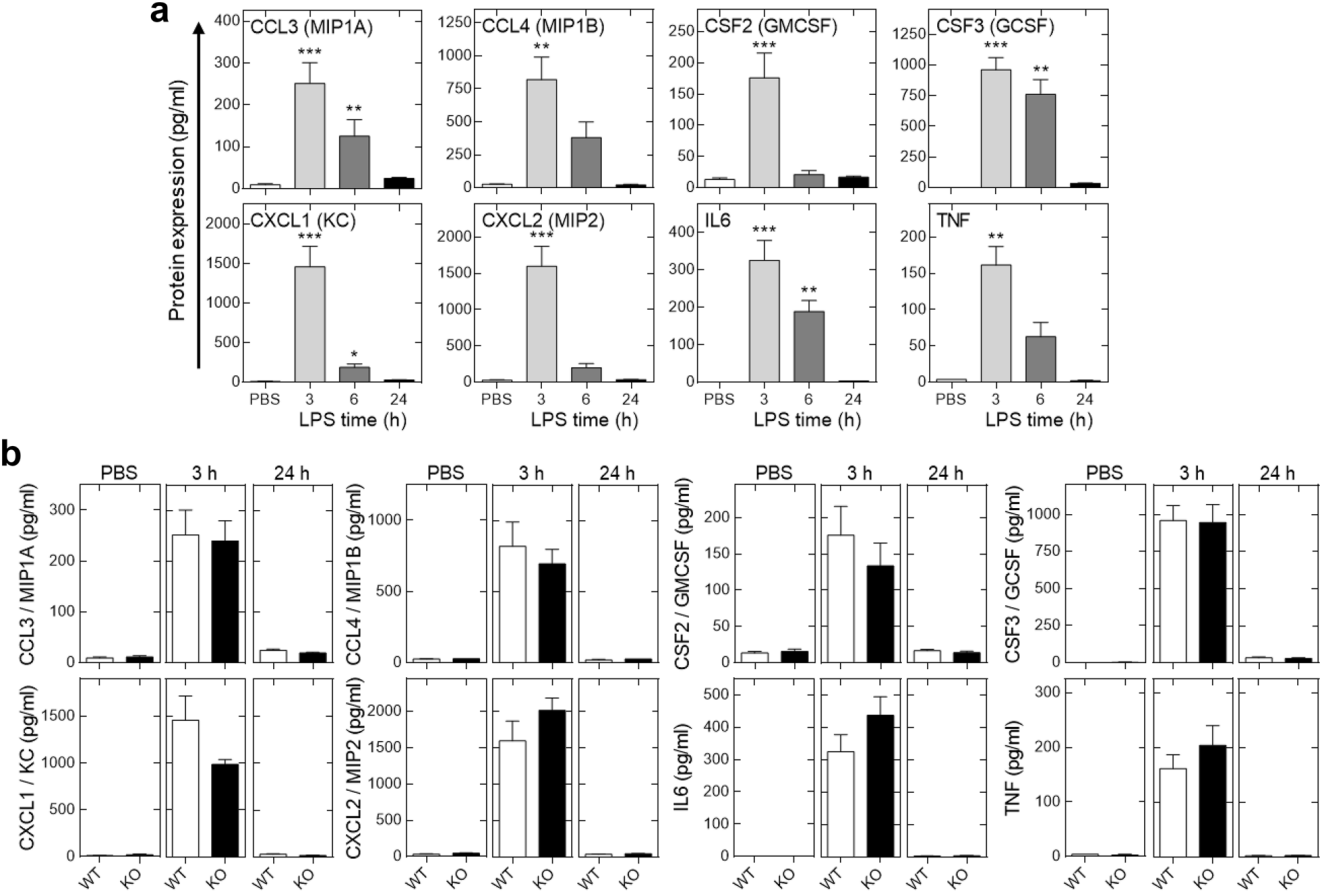
Effect of LPS and *Rgs2* deficiency on cytokine and chemokine expression in the BAL fluid. Wild type or *Rgs2*^−*/*−^ mice were exposed to aerosolized PBS (20 ml) or LPS from *E. coli* 0127:B8 (20 ml at 150 μg/ml) for 30 min. BAL fluid was prepared 3, 6 or 24 h after LPS challenge, as indicated. Following centrifugation to remove cells, the supernatants were analyzed by Luminex. **a** Expression of those mediators showing LPS-induced expression of over 100 pg/ml in wild type animals is shown. Data from wild type animals (*N* = 6–8) is plotted in pg/ml as mean ± SE. Significance relative to PBS control was assessed by ANOVA with a Dunn’s post test. *P ≤ 0.05, **P ≤ 0.01, ***P ≤ 0.001. **b** Expression of cytokines and chemokines was compared between wild type (WT) and *Rgs2*^−*/*−^ (KO) animals 3 h following PBS exposure or 3 and 24 h following LPS exposure. Wild type, *N* = 6, 8 and 8 (respectively), or *Rgs2*^−*/*−^ 3, 8 and 8 (respectively). Significance was tested by Mann–Whitney U test. No significant differences between wild type or *Rgs2*^−*/*−^ were detected

As described for Fig. 5, wild type or *Rgs2*^−*/*−^ mice were exposed to aerosolized PBS (20 ml) or LPS from *E. coli* 0127:B8 (20 ml at 150 μg/ml) for 30 min. BAL fluid was prepared 3, 6 or 24 h after LPS challenge, as indicated. Following centrifugation to remove cells, the supernatants were analyzed by Luminex. Those mediators showing LPS-induced expression of < 5 pg/ml in wild type or *Rgs2*^−*/*−^ animals are listed in A. Mediators showing LPS-induced expression < 100 pg/ml in wild type or *Rgs2*^−*/*−^ animals are shown in B. Data (*N* = 6–8) are provided in pg/ml as mean ± SE. Significance between PBS control and the 3 and 24 h post-LPS exposure was assessed by nonparametric ANOVA with a Dunn’s post test. **P ≤ 0.01, ***P ≤ 0.001. Significance between wild type and *Rgs2*^−*/*−^ animals at each time post-LPS treatment was tested by Mann–Whitney U test. ^#^*P* ≤ 0.05, ^###^*P* ≤ 0.001

The most highly expressed and LPS-induced cytokines and chemokines, here CCL3, CCL4, the colony-stimulate factors (CSFs), CSF2 & CSF3, and CXCL1, CXCL2, IL6 and tumor necrosis factor-α (TNF) were all significantly induced 3 h post-LPS exposure and this effect had largely disappeared 24 h post-exposure (Fig. 5a). In each case, there was no significant effect of *Rgs2* loss either at baseline or following LPS exposure (Fig. 5b). Following RNA extraction from whole lung, PCR was used to examine the mRNA expression of CCL3, CCL4, CCL11, CCL20, CSF2, CXCL1, CXCL2, CXCL10, IL6, and TNF. In each case, there was a marked induction of each mRNA following LPS exposure, but there were no significant effects due to the loss of *Rgs2* (data not shown).

## Discussion

As may occur with acute bacterial infections, the current LPS exposure model produced a rapid onset neutrophilic inflammation of the airways that was associated with transient AHR. Robust neutrophil recruitment into the lungs and BAL fluid was evident 3 h post LPS-exposure, presumably due to the rapid production of pro-neutrophilic/pro-inflammatory chemokines and cytokines observed 3 h following LPS exposure. These findings are consistent with prior studies using aerosolized or intra-nasal LPS administration [37, 41–45]. The current analysis found no evidence of PAS staining in the lungs, suggesting that mucus hypersecretion was not a feature of the acute response to LPS. There was also no evidence of LPS-induced airway remodelling. This is consistent with the acute nature of the model, but, as reported elsewhere, may occur at later times post-exposure [45]. LPS-induced AHR was transient, with marked increases in MCh-induced reactivity observed 3 h following LPS exposure, being largely resolved at 24 h. This result was slightly unexpected as the rapid accumulation of neutrophils into the BAL fluid at 3 h was maintained at both 6 and 24 h post-LPS exposure. Nevertheless, overall lung inflammation, as evidenced by H&E staining, was maximal around 3 h and by 6 h, and certainly 24 h, post-LPS exposure was resolving in the wild type animals. This is consistent with the expression of major inflammatory cytokines (CSF2, CSF3, IL6, TNF) and key neutrophil chemoattractants, including CXCL1 and CXCL2. These were all highly expressed in the lung and BAF fluid at 3 h, but by 24 h post-LPS exposure, expression had declined. Of relevance are possible effects of anesthetics, including pentobarbital and ketamine. These may directly, and/or indirectly, impact on inflammation [46]. While used prior to lung function analysis and/or lung removal, the time available to modulate existing LPS-induced inflammation is short and, therefore, unlikely to materially effect outcomes. Use of these compounds is also common to each study arm and therefore controlled within the study design.

In the absence of inflammatory stimulus, *Rgs2* loss produced a marked AHR apparent on MCh challenge [26, 28]. The current study extends these data by showing that LPS-induced reductions in lung function were further exacerbated in animals lacking *Rgs2*. Thus, 3 h post-LPS exposure, airways resistance induced by MCh was significantly greater in *Rgs2* knock-out animals. Possibly more striking was that 24 h post-LPS exposure, lung function in the wild type animals was recovering, whereas in the *Rgs2*^−*/*−^ animals, MCh-induced resistance remained elevated. Furthermore, at 24 h, *Rgs2* knock-out animals displayed a markedly and significantly reduced compliance compared to wild type animals. Such data, together with those of prior studies, confirm that the *Rgs2* gene is not only bronchoprotective in the absence of airway inflammation, but is also bronchoprotective in chronic airway inflammation induced by HDM, ovalbumin, IL13 and, as now reported, in acute neutrophilic inflammation [26–28, 47]. Interestingly, not only was RGS2 bronchoprotective, but the effect of *Rgs2* loss on lung compliance at 24 h post-LPS exposure suggests roles for RGS2 in assisting with resolution, or protection, post-exposure. What these mechanisms could be are unclear, but may relate to *Rgs2* loss enhancing the profibrotic and remodelling effects of receptors, including PARs [16, 48–50]. Such effects may become more relevant in chronic neutrophilic inflammatory diseases, as caused by cigarette and smoke inhalation [51]. However, as noted, no effects of *Rgs2* deficiency were apparent on remodelling and this would require investigation in longer-term models.

In terms of roles for *Rgs*2, in the absence of inflammatory stimulus, prior studies show no effect of *Rgs2* gene loss on lung inflammation or the recruitment of inflammatory cells into the BAL, when compared to wild type animals [26, 28]. In the current study, an effect of *Rgs2* deficiency on LPS-induced inflammation was hypothesized. However, when compared to wild type, no major change in the inflammatory response to LPS was noted in animals lacking *Rgs2*. In each case, lung inflammation and inflammatory cell recruitment, including neutrophils, to the BAL fluid were similar. Furthermore, expression of the most highly expressed cytokines and chemokines was similar in *Rgs2*^−*/*−^ and wild type animals. Such data do not support a major role, protective or otherwise, for RGS2 in LPS-induced airway inflammation and is consistent with a lack of the effect of *Rgs2* deletion on inflammatory indices in HDM- and IL13-induced inflammation [28, 47]. In this regard, LPS acts via TLR4 to induce inflammation [52]. As this pathway does not directly involve Gq-coupled GPCRs and primarily promotes expression of inflammatory mediators, such as TNF, via the activation of NF-κB and MAPKs [53], direct effects of RGS2 were not anticipated. Nevertheless, our original hypothesis was that neutrophilic inflammation, induced by LPS inhalation, may involve GPCRs that are regulated by RGS2. Numerous neutrophil-derived mediators act on GPCRs, many of which do couple via Gq [54]. The release such mediators is induced by LPS and roles for Gq-coupled GPCRs in accentuating, or further promoting, inflammatory responses were therefore expected. Similarly, numerous proteases are produced by neutrophils, and other inflammatory processes, and may cleave and activate PARs [55]. Both PAR1 and PAR2 are present on multiple cell types in the airways, in particular structural cells, including the epithelium and ASM, and can also be activated by neutrophil proteases, including neutrophil elastase [7, 55]. Indeed, PAR activation enhances expression of various inflammatory mediators [16, 19–21, 56, 57]. Similarly, PARs are implicated in various pro-inflammatory effects in vivo [7, 17, 18, 36, 55]. Thus, the current data showing no clear effect of *Rgs2* loss on inflammation, even 24 h post-exposure, requires explanation. One possibility may be that PARs do play a role in neutrophilic inflammation, but that these receptors, or their relevant downstream signaling pathways, for example. Gi, G12/13 or β-arrestin, unlike Gq, are not targeted by RGS2 and are therefore unaffected by *Rgs2* deficiency. However, while understanding of the selectivity of different RGS proteins for specific GPCRs is incomplete [58, 59], there is little suggestion of Gq-coupled GPCR selectivity for RGS2 and multiple studies indicate that, at least, PAR1 signalling is targeted by RGS2 [60–62].

Further explanations for the above data may come from a more detailed consideration of the roles for Gq-coupled GPCRs, such as PAR1 and PAR2, in inflammation. These are not simply “pro-inflammatory”, both pro- and anti-inflammatory effects are attributed to PARs [55, 63]. For example, agonism at Gq-coupled GPCRs leads to phospholipase A_2_ activation to induce prostaglandin (PG) production in epithelial cells [64]. PAR activation certainly promotes PGE_2_ release, which, in turn, can dampen expression of inflammatory cytokines and provide bronchoprotection [34, 63, 65–67]. Indeed, the PAR2-PGE_2_ axis was anti-inflammatory in an ovalbumin sensitized model of allergic airway inflammation [68]. Thus, RGS2 may well modulate Gq-coupled GPCR activity, but the effects of this could be both pro- and anti-inflammatory leading to little, or no, net effect due to *Rgs2* deletion.

Finally, while there was no clear effect of *Rgs2* knockout on the expression of highly expressed cytokines and chemokines, including CCL4, CCL4, CSF2, CSF3, CXCL1, CXCL2, IL6 and TNF, *Rgs2* loss significantly increased IL12B expression. Given a central role for IL12 in the development of Th1 responses, these data raise the possibility that RGS2 may play a regulatory role in the modulation of Th polarization. Indeed, neutrophil elastase promotes IL12 generation from LPS-stimulated macrophage via an apparently PAR2-dependent mechanism [69]. This could be enhanced in the absence of RGS2 or, possibly, abrogated by increased RGS2 expression. Similar effects may occur on dendritic cells, a known source of IL12 [70], which, like macrophage, are present in the lungs. Since, (1) IL12 and other Th1 gene polymorphisms are associated with severe asthma [71, 72]; and (2) in the context of allergen-induced inflammation and AHR, IL12 reduces Th2 responses, including AHR [70], further analysis of roles for RGS2 in T-helper cell programming/function is appropriate.

## Conclusions

The current data provide clear evidence for a bronchoprotective role of RGS2 in a murine model of acute neutrophilic inflammation. Not only was the presence of the wild type *Rgs2* gene protective from peak LPS-induced AHR, but *Rgs2* loss reduced the resolution of AHR 24 h post LPS exposure. This is potentially important in the context of human airway disease, where neutrophilic inflammation is notoriously hard to treat, and inhaled corticosteroids (ICSs) alone are often ineffective [1, 73]. The data, therefore support the concept that pharmacological interventions, which increase RGS2 expression, may be beneficial due to the protective effects of RGS2 on lung function [74]. Since ICS/long-acting β_2_-adrenoceptor agonist (LABA) combinations can synergistically enhance RGS2 expression, for example in human ASM [26], this is predicted to enhance bronchoprotection. The present study therefore supports the clinical use of therapies, such as ICS/LABA combination inhalers, that promote RGS2 expression to improve lung function in acute neutrophilic inflammation, such as exacerbations of asthma or chronic obstructive pulmonary disease [73, 74]. However, the data also indicate no clear anti-inflammatory role for RGS2 in acute inflammation. Thus, the continued search for more effective anti-inflammatory agents against neutrophilic disease is warranted. Finally, the finding that *Rgs2* loss promotes IL12B expression indicates a need to explore possible roles for RGS2 in the regulation of Th1 vs. Th2 programming.

## Additional file

**Additional file 1: Tables S1, S2.** Primers and probes used for genotyping. Primers used for qPCR.

## Abbreviations

AHR: airways hyperreactivity
ASM: airway smooth muscle
BAL: bronchoalveolar lavage
CCL: C-C motif chemokine ligand
CSF: colony-stimulating factor
CXCL: C-X-C motif chemokine ligand
GPCR: G protein-coupled receptor
H&E: hematoxylin and eosin
HDM: house dust mite
ICS: inhaled corticosteroid
IL: interleukin
LABA: long-acting β_2_-adrenoceptor agonist
LIF: leukemia inhibitory factor
LPS: lipopolysaccharide
MCh: methacholine
PAR: protease-activated receptor
PAS: periodic acid-schiff
PBS: phosphate buffered saline
PG: prostaglandin
RGS: regulator of G protein signaling
TNF: tumor necrosis factor-α

## References

[1] Barnes PJ (2008). Immunology of asthma and chronic obstructive pulmonary disease. Nat Rev Immunol.

[2] Penn RB, Benovic JL (2008). Regulation of heterotrimeric G protein signaling in airway smooth muscle. Proc Am Thorac Soc.

[3] Ammit AJ, Lazaar AL, Irani C, O’Neill GM, Gordon ND, Amrani Y, Penn RB, Panettieri RA (2002). Tumor necrosis factor-alpha-induced secretion of RANTES and interleukin-6 from human airway smooth muscle cells: modulation by glucocorticoids and beta-agonists. Am J Respir Cell Mol Biol.

[4] Holden NS, Rider CF, Bell MJ, Velayudhan J, King EM, Kaur M, Salmon M, Giembycz MA, Newton R (2010). Enhancement of inflammatory mediator release by beta(2)-adrenoceptor agonists in airway epithelial cells is reversed by glucocorticoid action. Br J Pharmacol.

[5] Jacquet A (2011). The role of innate immunity activation in house dust mite allergy. Trends Mol Med.

[6] Gregory LG, Lloyd CM (2011). Orchestrating house dust mite-associated allergy in the lung. Trends Immunol.

[7] Ramachandran R, Altier C, Oikonomopoulou K, Hollenberg MD (2016). Proteinases, their extracellular targets, and inflammatory signaling. Pharmacol Rev.

[8] Johnson JR, Wiley RE, Fattouh R, Swirski FK, Gajewska BU, Coyle AJ, Gutierrez-Ramos JC, Ellis R, Inman MD, Jordana M (2004). Continuous exposure to house dust mite elicits chronic airway inflammation and structural remodeling. Am J Respir Crit Care Med.

[9] Southam DS, Ellis R, Wattie J, Inman MD (2007). Components of airway hyperresponsiveness and their associations with inflammation and remodeling in mice. J Allergy Clin Immunol..

[10] Gregory LG, Causton B, Murdoch JR, Mathie SA, O’Donnell V, Thomas CP, Priest FM, Quint DJ, Lloyd CM (2009). Inhaled house dust mite induces pulmonary T helper 2 cytokine production. Clin Exp Allergy.

[11] Boitano S, Hoffman J, Flynn AN, Asiedu MN, Tillu DV, Zhang Z, Sherwood CL, Rivas CM, DeFea KA, Vagner J, Price TJ (2015). The novel PAR2 ligand C391 blocks multiple PAR2 signalling pathways in vitro and in vivo. Br J Pharmacol..

[12] Asokananthan N, Graham PT, Stewart DJ, Bakker AJ, Eidne KA, Thompson PJ, Stewart GA (2002). House dust mite allergens induce proinflammatory cytokines from respiratory epithelial cells: the cysteine protease allergen, Der p 1, activates protease-activated receptor (PAR)-2 and inactivates PAR-1. J Immunol.

[13] Ebeling C, Lam T, Gordon JR, Hollenberg MD, Vliagoftis H (2007). Proteinase-activated receptor-2 promotes allergic sensitization to an inhaled antigen through a TNF-mediated pathway. J Immunol.

[14] Arizmendi NG, Abel M, Mihara K, Davidson C, Polley D, Nadeem A, El MT, Gilmore BF, Walker B, Gordon JR, Hollenberg MD, Vliagoftis H (2011). Mucosal allergic sensitization to cockroach allergens is dependent on proteinase activity and proteinase-activated receptor-2 activation. J Immunol.

[15] Davidson CE, Asaduzzaman M, Arizmendi NG, Polley D, Wu Y, Gordon JR, Hollenberg MD, Cameron L, Vliagoftis H (2013). Proteinase-activated receptor-2 activation participates in allergic sensitization to house dust mite allergens in a murine model. Clin Exp Allergy.

[16] Schuliga M, Royce SG, Langenbach S, Berhan A, Harris T, Keenan CR, Stewart AG (2016). The coagulant factor Xa induces protease-activated receptor-1 and annexin A2-dependent airway smooth muscle cytokine production and cell proliferation. Am J Respir Cell Mol Biol.

[17] Williams JC, Lee RD, Doerschuk CM, Mackman N (2011). Effect of PAR-2 deficiency in mice on KC expression after intratracheal LPS administration. J Signal Transduct.

[18] Antoniak S, Owens AP, Baunacke M, Williams JC, Lee RD, Weithauser A, Sheridan PA, Malz R, Luyendyk JP, Esserman DA, Trejo J, Kirchhofer D, Blaxall BC, Pawlinski R, Beck MA, Rauch U, Mackman N (2013). PAR-1 contributes to the innate immune response during viral infection. J Clin Invest.

[19] Mihara K, Ramachandran R, Renaux B, Saifeddine M, Hollenberg MD (2013). Neutrophil elastase and proteinase-3 trigger G protein-biased signaling through proteinase-activated receptor-1 (PAR1). J Biol Chem.

[20] Zhao P, Lieu T, Barlow N, Sostegni S, Haerteis S, Korbmacher C, Liedtke W, Jimenez-Vargas NN, Vanner SJ, Bunnett NW (2015). Neutrophil elastase activates protease-activated receptor-2 (PAR2) and transient receptor potential vanilloid 4 (TRPV4) to cause inflammation and pain. J Biol Chem.

[21] Muley MM, Reid AR, Botz B, Bolcskei K, Helyes Z, McDougall JJ (2016). Neutrophil elastase induces inflammation and pain in mouse knee joints via activation of proteinase-activated receptor-2. Br J Pharmacol.

[22] Koga H, Miyahara N, Fuchimoto Y, Ikeda G, Waseda K, Ono K, Tanimoto Y, Kataoka M, Gelfand EW, Tanimoto M, Kanehiro A (2013). Inhibition of neutrophil elastase attenuates airway hyperresponsiveness and inflammation in a mouse model of secondary allergen challenge: neutrophil elastase inhibition attenuates allergic airway responses. Respir Res.

[23] Kimple AJ, Bosch DE, Giguere PM, Siderovski DP (2011). Regulators of G-protein signaling and their Galpha substrates: promises and challenges in their use as drug discovery targets. Pharmacol Rev.

[24] Xie Z, Chan EC, Druey KM (2016). R4 regulator of G protein signaling (RGS) proteins in inflammation and immunity. AAPS J.

[25] Damera G, Druey KM, Cooper PR, Krymskaya VP, Soberman RJ, Amrani Y, Hoshi T, Brightling CE, Panettieri RA (2012). An RGS4-mediated phenotypic switch of bronchial smooth muscle cells promotes fixed airway obstruction in asthma. PLoS ONE.

[26] Holden NS, Bell MJ, Rider CF, King EM, Gaunt DD, Leigh R, Johnson M, Siderovski DP, Heximer SP, Giembycz MA, Newton R (2011). beta2-Adrenoceptor agonist-induced RGS2 expression is a genomic mechanism of bronchoprotection that is enhanced by glucocorticoids. Proc Natl Acad Sci USA.

[27] Xie Y, Jiang H, Nguyen H, Jia S, Berro A, Panettieri RA, Wolff DW, Abel PW, Casale TB, Tu Y (2012). Regulator of G protein signaling 2 is a key modulator of airway hyperresponsiveness. J Allergy Clin Immunol.

[28] George T, Bell M, Chakraborty M, Siderovski DP, Giembycz MA, Newton R (2017). Protective roles for RGS2 in a mouse model of house dust mite-induced airway inflammation. PLoS ONE.

[29] Balenga NA, Jester W, Jiang M, Panettieri RA, Druey KM (2014). Loss of regulator of G protein signaling 5 promotes airway hyperresponsiveness in the absence of allergic inflammation. J Allergy Clin Immunol.

[30] Yang Z, Cooper PR, Damera G, Mukhopadhyay I, Cho H, Kehrl JH, Panettieri RA, Druey KM (2011). Beta-agonist-associated reduction in RGS5 expression promotes airway smooth muscle hyper-responsiveness. J Biol Chem.

[31] Leigh R, Mostafa MM, King EM, Rider CF, Shah S, Dumonceaux C, Traves SL, McWhae A, Kolisnik T, Kooi C, Slater DM, Kelly MM, Bieda M, Miller-Larsson A, Newton R (2016). An inhaled dose of budesonide induces genes involved in transcription and signaling in the human airways: enhancement of anti- and proinflammatory effector genes. Pharma Res Per.

[32] Holden NS, George T, Rider CF, Chandrasekhar A, Shah S, Kaur M, Johnson M, Siderovski DP, Leigh R, Giembycz MA, Newton R (2014). Induction of regulator of G-protein signaling 2 expression by long-acting beta2-adrenoceptor agonists and glucocorticoids in human airway epithelial cells. J Pharmacol Exp Ther.

[33] Knight DA, Lim S, Scaffidi AK, Roche N, Chung KF, Stewart GA, Thompson PJ (2001). Protease-activated receptors in human airways: upregulation of PAR-2 in respiratory epithelium from patients with asthma. J Allergy Clin Immunol.

[34] Asokananthan N, Graham PT, Fink J, Knight DA, Bakker AJ, McWilliam AS, Thompson PJ, Stewart GA (2002). Activation of protease-activated receptor (PAR)-1, PAR-2, and PAR-4 stimulates IL-6, IL-8, and prostaglandin E2 release from human respiratory epithelial cells. J Immunol.

[35] Jose RJ, Williams AE, Mercer PF, Sulikowski MG, Brown JS, Chambers RC (2015). Regulation of neutrophilic inflammation by proteinase-activated receptor 1 during bacterial pulmonary infection. J Immunol.

[36] Schuliga M (2015). The inflammatory actions of coagulant and fibrinolytic proteases in disease. Mediators Inflamm.

[37] Lefort J, Motreff L, Vargaftig BB (2001). Airway administration of *Escherichia coli* endotoxin to mice induces glucocorticosteroid-resistant bronchoconstriction and vasopermeation. Am J Respir Cell Mol Biol.

[38] Oliveira-Dos-Santos AJ, Matsumoto G, Snow BE, Bai D, Houston FP, Whishaw IQ, Mariathasan S, Sasaki T, Wakeham A, Ohashi PS, Roder JC, Barnes CA, Siderovski DP, Penninger JM (2000). Regulation of T cell activation, anxiety, and male aggression by RGS2. Proc Natl Acad Sci USA.

[39] Heximer SP, Knutsen RH, Sun X, Kaltenbronn KM, Rhee MH, Peng N, Oliveira-dos-Santos A, Penninger JM, Muslin AJ, Steinberg TH, Wyss JM, Mecham RP, Blumer KJ (2003). Hypertension and prolonged vasoconstrictor signaling in RGS2-deficient mice. J Clin Invest.

[40] Shalaby KH, Gold LG, Schuessler TF, Martin JG, Robichaud A (2010). Combined forced oscillation and forced expiration measurements in mice for the assessment of airway hyperresponsiveness. Respir Res.

[41] Puljic R, Benediktus E, Plater-Zyberk C, Baeuerle PA, Szelenyi S, Brune K, Pahl A (2007). Lipopolysaccharide-induced lung inflammation is inhibited by neutralization of GM-CSF. Eur J Pharmacol.

[42] Chignard M, Balloy V (2000). Neutrophil recruitment and increased permeability during acute lung injury induced by lipopolysaccharide. Am J Physiol Lung Cell Mol Physiol.

[43] Campanholle G, Landgraf RG, Borducchi E, Semedo P, Wang PH, Amano MT, Russo M, Pacheco-Silva A, Jancar S, Camara NO (2010). Bradykinin inducible receptor is essential to lipopolysaccharide-induced acute lung injury in mice. Eur J Pharmacol.

[44] Roos AB, Berg T, Ahlgren KM, Grunewald J, Nord M (2014). A method for generating pulmonary neutrophilia using aerosolized lipopolysaccharide. J Vis Exp.

[45] de Souza Xavier Costa N, Ribeiro Júnior G, Dos Santos Alemany AA, Belotti L, Zati DH, Frota Cavalcante M, Matera Veras M, Ribeiro S, Kallás EG, Nascimento Saldiva PH, Dolhnikoff M, Ferraz da Silva LF (2017). Early and late pulmonary effects of nebulized LPS in mice: an acute lung injury model. PLoS ONE..

[46] Cruz FF, Rocco PR, Pelosi P (2017). Anti-inflammatory properties of anesthetic agents. Crit Care.

[47] Jiang H, Xie Y, Abel PW, Wolff DW, Toews ML, Panettieri RA, Casale TB, Tu Y (2015). Regulator of G-protein signaling 2 repression exacerbates airway hyper-responsiveness and remodeling in asthma. Am J Respir Cell Mol Biol.

[48] Akers IA, Parsons M, Hill MR, Hollenberg MD, Sanjar S, Laurent GJ, McAnulty RJ (2000). Mast cell tryptase stimulates human lung fibroblast proliferation via protease-activated receptor-2. Am J Physiol Lung Cell Mol Physiol.

[49] Momen A, Afroze T, Sadi AM, Khoshbin A, Zhang H, Choi J, Gu S, Zaidi SH, Heximer SP, Husain M (2014). Enhanced proliferation and altered calcium handling in RGS2-deficient vascular smooth muscle cells. J Recept Signal Transduct Res.

[50] Allard B, Bara I, Gilbert G, Carvalho G, Trian T, Ozier A, Gillibert-Duplantier J, Ousova O, Maurat E, Thumerel M, Quignard JF, Girodet PO, Marthan R, Berger P (2014). Protease activated receptor-2 expression and function in asthmatic bronchial smooth muscle. PLoS ONE.

[51] Roos AB, Stampfli MR (2017). Targeting Interleukin-17 signalling in cigarette smoke-induced lung disease: mechanistic concepts and therapeutic opportunities. Pharmacol Ther.

[52] Togbe D, Schnyder-Candrian S, Schnyder B, Couillin I, Maillet I, Bihl F, Malo D, Ryffel B, Quesniaux VF (2006). TLR4 gene dosage contributes to endotoxin-induced acute respiratory inflammation. J Leukoc Biol.

[53] Togbe D, Schnyder-Candrian S, Schnyder B, Doz E, Noulin N, Janot L, Secher T, Gasse P, Lima C, Coelho FR, Vasseur V, Erard F, Ryffel B, Couillin I, Moser R (2007). Toll-like receptor and tumour necrosis factor dependent endotoxin-induced acute lung injury. Int J Exp Pathol.

[54] Tintinger GR, Anderson R, Feldman C (2013). Pharmacological approaches to regulate neutrophil activity. Semin Immunopathol.

[55] Vergnolle N (2009). Protease-activated receptors as drug targets in inflammation and pain. Pharmacol Ther.

[56] Vliagoftis H, Schwingshackl A, Milne CD, Duszyk M, Hollenberg MD, Wallace JL, Befus AD, Moqbel R (2000). Proteinase-activated receptor-2-mediated matrix metalloproteinase-9 release from airway epithelial cells. J Allergy Clin Immunol.

[57] Vliagoftis H, Befus AD, Hollenberg MD, Moqbel R (2001). Airway epithelial cells release eosinophil survival-promoting factors (GM-CSF) after stimulation of proteinase-activated receptor 2. J Allergy Clin Immunol.

[58] Kimple AJ, Soundararajan M, Hutsell SQ, Roos AK, Urban DJ, Setola V, Temple BR, Roth BL, Knapp S, Willard FS, Siderovski DP (2009). Structural determinants of G-protein alpha subunit selectivity by regulator of G-protein signaling 2 (RGS2). J Biol Chem.

[59] Heximer SP (2013). A “new twist” on RGS protein selectivity. Structure.

[60] Karakoula A, Tovey SC, Brighton PJ, Willars GB (2008). Lack of receptor-selective effects of either RGS2, RGS3 or RGS4 on muscarinic M3- and gonadotropin-releasing hormone receptor-mediated signalling through G alpha q/11. Eur J Pharmacol.

[61] Chen B, Siderovski DP, Neubig RR, Lawson MA, Trejo J (2014). Regulation of protease-activated receptor 1 signaling by the adaptor protein complex 2 and R4 subfamily of regulator of G protein signaling proteins. J Biol Chem.

[62] Ghil S, McCoy KL, Hepler JR (2014). Regulator of G protein signaling 2 (RGS2) and RGS4 form distinct G protein-dependent complexes with protease activated-receptor 1 (PAR1) in live cells. PLoS ONE.

[63] Peters T, Henry PJ (2009). Protease-activated receptors and prostaglandins in inflammatory lung disease. Br J Pharmacol.

[64] Newton R, Eddleston J, Haddad E, Hawisa S, Mak J, Lim S, Fox AJ, Donnelly LE, Chung KF (2002). Regulation of kinin receptors in airway epithelial cells by inflammatory cytokines and dexamethasone. Eur J Pharmacol.

[65] Slater DM, Astle S, Woodcock N, Chivers JE, de Wit NC, Thornton S, Vatish M, Newton R (2006). Anti-inflammatory and relaxatory effects of prostaglandin E2 in myometrial smooth muscle. Mol Hum Reprod.

[66] Knight DA, Stewart GA, Thompson PJ (1995). Prostaglandin E2, but not prostacyclin inhibits histamine-induced contraction of human bronchial smooth muscle. Eur J Pharmacol.

[67] Lan RS, Knight DA, Stewart GA, Henry PJ (2001). Role of PGE(2) in protease-activated receptor-1, -2 and -4 mediated relaxation in the mouse isolated trachea. Br J Pharmacol.

[68] De Campo BA, Henry PJ (2005). Stimulation of protease-activated receptor-2 inhibits airway eosinophilia, hyperresponsiveness and bronchoconstriction in a murine model of allergic inflammation. Br J Pharmacol.

[69] Yamaguchi R, Yamamoto T, Sakamoto A, Narahara S, Sugiuchi H, Yamaguchi Y (2016). Neutrophil elastase enhances IL-12p40 production by lipopolysaccharide-stimulated macrophages via transactivation of the PAR-2/EGFR/TLR4 signaling pathway. Blood Cells Mol Dis.

[70] Lewkowich IP, Lajoie S, Stoffers SL, Suzuki Y, Richgels PK, Dienger K, Sproles AA, Yagita H, Hamid Q, Wills-Karp M (2013). PD-L2 modulates asthma severity by directly decreasing dendritic cell IL-12 production. Mucosal Immunol.

[71] Randolph AG, Lange C, Silverman EK, Lazarus R, Silverman ES, Raby B, Brown A, Ozonoff A, Richter B, Weiss ST (2004). The IL12B gene is associated with asthma. Am J Hum Genet.

[72] Li X, Hawkins GA, Ampleford EJ, Moore WC, Li H, Hastie AT, Howard TD, Boushey HA, Busse WW, Calhoun WJ, Castro M, Erzurum SC, Israel E, Lemanske RF, Szefler SJ, Wasserman SI, Wenzel SE, Peters SP, Meyers DA, Bleecker ER (2013). Genome-wide association study identifies TH1 pathway genes associated with lung function in asthmatic patients. J Allergy Clin Immunol.

[73] Newton R, Giembycz MA (2016). Understanding how long-acting beta2 -adrenoceptor agonists enhance the clinical efficacy of inhaled corticosteroids in asthma—an update. Br J Pharmacol.

[74] Newton R (2018). Regulators of G-protein signaling as asthma therapy?. Am J Respir Cell Mol Biol.

