# Supplementary material for: A bronchoprotective role for Rgs2 in a murine model of lipopolysaccharide-induced airways inflammation
Source: Allergy Asthma Clin Immunol. 2018 Oct 1;14:40. doi: 10.1186/s13223-018-0266-5 (PMC6166284; doi:10.1186/s13223-018-0266-5)
Supplement: Supplementary file 1 — Additional file 1: Tables S1, S2. Primers and probes used for genotyping. Primers used for qPCR. [file 13223_2018_266_MOESM1_ESM.pdf]

Additional file 1: Tables S1 and S2

Table S1: Primers and Probes used for genotyping

Primers sequences (5′- 3′) are shown for the wild type (WT) and *Rgs2*$^{-/-}$ knockout (KO) genotyping primers and Taqman probes. The probes were synthesized by Applied Biosystems and the primers were synthesised by the DNA synthesis lab at the University of Calgary.

Type	Name	Sequences
WT gene probe	WTP2mRGS2	VIC-CTGTAGTCCTAAGAACG-MGB
KO gene probe	KOP2mRGS2	6FAM-ATCCCCCAATTCTACCG-MGB
WT Forward primer	WTF4 mRGS2	GTTTACTGTGTGCAAGGGTGTTG
WT Reverse primer	WTR3 mRGS2	CAGTTTCAGTGATACGTGACACACTAG
KO Forward primer	KOF4 mRGS2	CAGACTGCCTTGGGAAAAGA
KO Reverse primer	KOR3 mRGS2	CAGTTTCAGTGATACGTGACACACTAG

Table S2: Primers used for qPCR analysis

Forward (F) and reverse (R) primer sequences (5' – 3′) are shown in addition to the accession number for each gene. For genes with more than one splice variant, primers were designed to pick up all variants. All primers were designed using Primer Express software (Applied Biosystems) and were synthesised by the DNA synthesis lab at the University of Calgary.

Target gene	Accession Number	Primer Sequences
CCL3	NM_011337.2	F: TCTTCTCAGCGCCATATGGA<br>R: TCCGGCTGTAGGAGAAGCA
CCL4	NM_013652.2	F: CAGCACCAATGGGCTCTGA<br>R: TGCCGGGAGGTGTAAGAGAA
CCL5	NM_013653.3	F: TGCCCACGTCAAGGAGTATTT<br>R: ACTTCTTCTCTGGGTTGGCAC
CCL11	NM_011330.3	F: ATCCCAACTTCCTGCTGCTTT<br>R: AGATCTCTTTGCCCAACCTGG
CCL20	NM_016960.2<br>NM_001159738.1	F: GTGGGTTTCACAAGACAGATG<br>R: TTTTCACCCAGTTCTGCTTTG
CXCL1	NM_008176.3	F: CAATGAGCTGCGCTGTCAGT<br>R: CTGGATGTTCTTGAGGTGAATCC
CXCL2	NM_009140.2	F: TCAAGAACATCCAGAGCTTGAG<br>R: TTCAGGGTCAAGGCAAACTT
CXCL10	NM_021274.2	F: CCAAGTGCTGCCGTCATTTT<br>R: TTCAAGCTTCCCTATGGCCC

GAPDH	NM_011339.2	F: TCGAGACCA TTTACTGCAACAGA<br>R: TTGGGCCAACAGTAGCCTTC
CSF2	NM_001289726.1<br>NM_008084.3	F: AGCCCATCACCATCTTCCAG<br>R: GATGACCCTTTTGGCTCCAC
IFNG	NM_009969.4	F: TCAAAGAAGCCCTGAACCTCC<br>R: GTGAAATTGCCCCGTAGACC
IL6	NM_008337.4	F: CCACGGCACAGTCATTGAAA<br>R: CTGCAGGATTTTCATGTCACCA
TNF	NM_031168.2<br>NM_001314054.1	F: TGTTCTCTGGGAAATCGTGGA<br>R: TGCAAGTGCATCATCGTTGTTC
